# Protein Posttranslational Modification in Stemness Remodeling and Its Emerging Role as a Novel Therapeutic Target in Gastrointestinal Cancers

**DOI:** 10.3390/ijms24119173

**Published:** 2023-05-24

**Authors:** Yifei Wang, Man Tong

**Affiliations:** School of Biomedical Sciences, The Chinese University of Hong Kong, Hong Kong SAR, China; yifeiwang@cuhk.edu.hk

**Keywords:** posttranslational modification, stemness, cancer stem cells, gastrointestinal cancers

## Abstract

The posttranslational modifications (PTMs) of proteins, as critical mechanisms for protein regulation, are well known to enhance the functional diversity of the proteome and dramatically participate in complicated biological processes. Recent efforts in the field of cancer biology have illustrated the extensive landscape of PTMs and their crosstalk with a wide range of pro-tumorigenic signaling pathways that decisively contribute to neoplastic transformation, tumor recurrence, and resistance to oncotherapy. Cancer stemness is an emerging concept that maintains the ability of tumor cells to self-renew and differentiate and has been recognized as the root of cancer development and therapy resistance. In recent years, the PTM profile for modulating the stemness of various tumor types has been identified. This breakthrough has shed light on the underlying mechanisms by which protein PTMs maintain cancer stemness, initiate tumor relapse, and confer resistance to oncotherapies. This review focuses on the latest knowledge of protein PTMs in reprogramming the stemness of gastrointestinal (GI) cancer. A deeper understanding of abnormal PTMs in specific proteins or signaling pathways provides an opportunity to specifically target cancer stem cells and highlights the clinical relevance of PTMs as potential biomarkers and therapeutic targets for patients with GI malignancies.

## 1. Introduction

Globally, gastrointestinal (GI) cancers, including liver, colorectal, gastric, pancreatic, and esophageal cancers, are the most common types of lethal malignancy that occur in the gastrointestinal tract. These cancers are caused by various risk factors, leading to alterations in the molecular networks across multiple levels of the genome, transcriptome, proteome, metabolome, and interactome [1,2]. Despite recent advances in their early diagnosis and treatment, GI cancers remain a major public health concern, with an estimated 4,975,378 new cases and 3,524,932 related deaths expected in 2020, accounting for more than one third of total cancer mortality [3]. Thus, an exploration of GI cancer biology is urgently needed.

Posttranslational modifications (PTMs) are covalent protein-processing events that significantly affect the structure and dynamics of proteins, thereby influencing the protein characteristics. PTMs involve proteolytic cleavage and adding a modifying group, such as acetyl, phosphoryl, glycosyl, and methyl, to amino acids [4]. This process is extremely important for the regulation of protein functions by significantly affecting the structure and dynamics of these proteins, and is therefore critical for numerous biological and pathological processes, including cancer stemness and tumor progression. Over the decades, there has been a growing interest in the concept of cancer stem cells (CSCs, also known as tumor-initiating cells (TILs)), which are a subpopulation of cells within the tumor bulk and are now known to be the driving force behind malignancy and disease progression in a variety of cancers [5]. Recent studies have demonstrated the crucial role of protein PTMs in regulating cancer stemness and tumor progression across various hematological and solid cancers, including GI malignancies [6]. Moreover, aberrant protein PTMs are responsible for the acquisition of therapeutic resistance by affecting cancer stemness [7,8,9,10,11,12,13]. Therefore, understanding the underlying mechanism by which CSCs acquire stemness properties through PTMs can facilitate the specific targeting of CSCs. The identification of the specific proteins and modifications that regulate cancer stemness could open up new avenues for the development of targeted therapies that can selectively eliminate CSCs and improve patient outcomes. This review provides new insights into diverse PTMs for remodeling the stemness in GI cancers, with a particular emphasis on protein phosphorylation, glycosylation, methylation, and acetylation. Additionally, we will discuss the potential clinical implications of targeting the PTMs in GI CSCs for cancer treatment.

## 2. Overview of Protein PTMs

**Figure 1:**
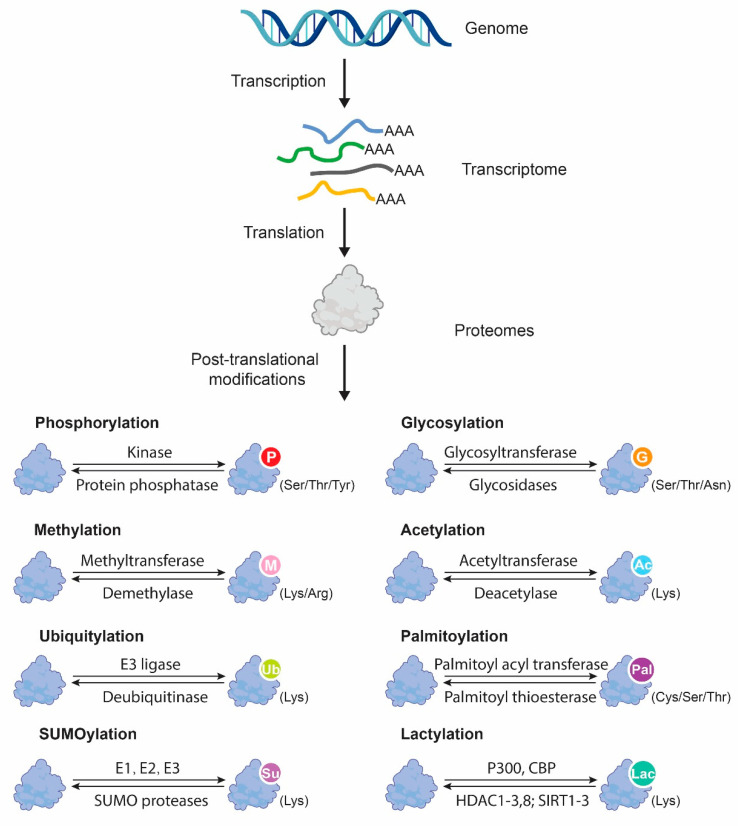
Summary of the eight most common types of protein post-translational modifications.

Protein PTMs are chemical alterations that involve introducing structural changes into existing proteins following protein synthesis [4]. According to their occurrence at specific motifs in a protein sequence, many different types of enzymatic PTMs have been identified, including chemical groups (e.g., phosphorylation and acetylation), glycans (glycosylation), or polypeptides (ubiquitination and SUMOylation) [14]. Each type of PTM is catalyzed by its specific enzymes or pathways and exhibits a unique functional effect on its targeted proteins (Figure 1). To date, enormous proteoforms are known to be produced by more than 200 known PTMs; hence, they contribute to a wide range of protein characteristics and behaviors [15]. For example, phosphorylation, as one of the most common PTMs, has been demonstrated to affect protein activity, stability, and localization [16]. Acetylation and methylation are reported to influence the interaction between proteins, DNA binding, and gene expression, while glycosylation is responsible for protein folding, trafficking, and stabilization [17]. Ubiquitination is a type of PTM that targets proteins for degradation by the proteasome and can also regulate protein activity and localization [18]. Furthermore, protein PTMs can occur in both histones and non-histone proteins, as histone PTMs mainly control epigenomic modification by regulating the gene transcription, while non-histone PTMs play a pivotal role in modulating protein functions such as protein stability, activity, localization, and their interactions with other molecules [19].

Under normal conditions, PTMs participate in a variety of biological processes, such as cell signaling transduction and cellular metabolism [20]. However, dysregulated PTMs have been shown to facilitate the development and progression of many diseases, including cancer, neurodegenerative diseases, and metabolic disorders [7,8,9]. In cancers, PTMs have been shown to regulate various oncoproteins and tumor suppressors, as well as control several key signaling pathways involved in tumor growth, invasion, and metastasis [21]. Moreover, PTMs also play essential roles in remodeling the tumor microenvironment, such as the remodeling of the extracellular matrix (ECM) and immune modulation [22].

## 3. Cancer Stemness

Cancer heterogeneity is known to be attributed to different cell subsets within the tumor bulk, in which subpopulations of cancer cells possessing stem-like properties, including self-renewing and differentiating abilities, have been termed as CSCs [23]. Surface markers and unique CSCs features have emerged to characterize CSCs in multiple solid cancers, including GI, breast, lung, kidney, brain, and prostate cancers and melanoma [24]. CSCs are not only responsible for tumor initiation, progression, enhanced angiogenesis, and metastasis, but also contribute to tumor immune evasion and resistance to conventional chemotherapy and radiotherapy, in support of tumorigenic phenotypes [23,25]. Targeting CSCs therefore represents a reliable strategy for eradicating the bulk of the tumor. In recent years, significant efforts have been put forward to analyze the altered transcriptomes, epigenomes, and proteomes of CSCs in comparison to non-CSCs. Extensive research findings have revealed that the molecular mechanisms that regulate the cancer stemness in GI cancers are complex and involve various signaling pathways, such as Wnt/β-catenin, Notch, and TGF-β signaling [26]. These pathways are activated by a variety of factors, including growth factors, cytokines, and extracellular matrix proteins, and they play a key role in allowing for CSC self-renewal, survival, and differentiation [26]. In addition, transcription factors, such as Oct4, Sox2, and Nanog, have been shown to be overexpressed in CSCs and represent key regulators of stem cell self-renewal and differentiation [26]. Metabolic enzymes, including the enzymes involved in glycolysis and oxidative phosphorylation, are also critical for these CSC survival and self-renewal properties [27].

## 4. PTMs and Cancer Stemness in GI Malignancies

Altered protein PTMs have been extensively studied in cancer biology, with various modifications, such as phosphorylation, acetylation, methylation, glycosylation, ubiquitination, and SUMOylation, which have been shown to be involved in the regulation of cancer stemness in GI malignancies [28]. Specific PTMs can regulate the activity of critical proteins and pathways that promote stemness in cancer cells, including transcription factors and metabolic enzymes. In addition, PTMs can regulate the activity of the transcription factors and metabolic enzymes that are critical for this stemness in cancer cells. In this section, a detailed summary of our current knowledge about the modulatory effects of PTMs on non-histone proteins (Table 1) and histone proteins (Table 2) and their roles in regulating cancer stemness in the five most common types of GI cancers, including hepatocellular carcinoma (HCC), esophageal cancer (EC), gastric cancer (GC), colorectal cancer (CRC), and pancreatic cancer (PC), will be reviewed.

### 4.1. Protein Phosphorylation and Cancer Stemness

Protein phosphorylation is the most prevalent experimentally observed PTM and plays a pivotal role in modulating GI cancer stemness. The hyperactivation of pro-tumorigenic signaling pathways, such as PI3K/Akt, NF-kB, and Hippo/YAP signaling, relies on the sequential phosphorylation of their substrates and has been well documented to support the stemness properties in HCC, GC, and CRC [29,30,31]. Furthermore, aberrant protein phosphorylation can lead to the dysregulation of the signaling pathways that contribute to the maintenance of cancer stemness. For example, the Wnt/β-catenin signaling pathway is a critical stemness-regulating mechanism, in which the phosphorylation of β-catenin and other critical players is tightly controlled to direct their cellular localization, degradation, and activities [32]. Individual phosphorylated proteins have also been shown to modulate stemness in GI cancers. For instance, CD24-expressing HCC CSCs were found to elevate NANOG expression via the induction of STAT3 phosphorylation, leading to enhanced self-renewal and tumor growth [33]. Upon mitophagy inhibition, PINK1 phosphorylated p53 proteins at the serine-392 on mitochondria, which facilitated the translocation of p53 into the nucleus and its binding with the NANOG promoter to suppress OCT4- and SOX2-induced NANOG transactivation [34]. This resulted in a reduced proportion of liver CSCs, suggesting the crucial role of the p53 phosphorylation status in the maintenance of cancer stemness [34]. Additionally, YAP dephosphorylation is responsible for promoting the CSC characteristics of CRC that are potentiated by the stiff matrix [35]. The administration of an anti-integrin β1 antibody and FAK inhibitor effectively inactivated YAP and its downstream PI3K/Akt signaling, resulting in decreased cancer stemness markers CD133, ALDH-1, and LGR-5 [35]. Furthermore, the Akt-mediated phosphorylation of transcription factors OCT4 at threonine 235 (Thr235) is responsible for regulating the sphere formation and tumorigenesis in HCC cells, while the treatment of ITE, an agonist of the aryl hydrocarbon receptor (AhR), could diminish this Akt-mediated OCT4 phosphorylation [36]. Taken together, exploiting the regulatory effect of protein phosphorylation in GI cancer stemness might benefit patients diagnosed with these lethal diseases.

### 4.2. Protein Glycosylation and Cancer Stemness

An integrative analysis of multi-omics data revealed a causal relationship between glycosylation and the stemness properties in HCC [37]. Glycosylation-associated genes, including CAD, SLC51B, LGALS3, B3GAT3, and MT3, were closely associated with stemness markers in a publicly available database [37]. The knockdown of these genes suppressed cell proliferation and reduced the expression of CSC-related markers such as CD24, CD44, CD20, EpCAM, and FOXM1 [37]. CD133^+^ liver CSCs were characterized by increased sialylation [38]. A further investigation identified an elevation in the fetuin-A sialylation in doxorubicin (DXR)-treated HCC cells, suggesting the possible effect of sialylation on cancer stemness [39]. ST6Gal1, the enzyme that catalyzes tumor-associated α2,6-sialylation, has been consistently elevated across GI tumors and favors stemness properties, including increased CSC surface markers, enhanced spheroid growth, and the upregulation of stemness-associated transcription factors such as Sox9 and Slug [7,40]. As such, the depletion of ST6Gal1 led to decreased CD133^+^/ALDH1^+^ colon CSCs and an impaired tumor-initiating potential in PDAC MiaPaCa2 cells, as determined by in vivo limiting dilution assays [7,40]. In addition, hyperfucosylation has been implicated in the aggressive cancer stemness features in GI cancers [10]. Loong et al. demonstrated that the overexpression of fucosyltransferase 1 (FUT1) was highly correlated with aggressive tumor features and was responsible for enhanced cell viability and self-renewal [10]. Upon glucose restriction, the activation of the PERK/eIF2α/ATF4 axis transcriptionally upregulated FUT1, which increased α1,2-fucosylation, the membrane translocation/activation of CD147, ICAM-1, EGFR, and EPHA2, and further activated the Akt/mTOR/4EBP1 pathway [10]. Similarly, FUT9-mediated fucosylation also triggered the stemness reprogramming of CRC, as evidenced by an increase in the CSC marker expression (e.g., Sox2, ALDH, and CD44), tumor-sphere formation, and 5-FU resistance observed in high-FUT9-expressing murine colon adenocarcinoma MC38 cells, human CRC cells, and primary CRC tumors [9]. 

In addition to sialylation and fucosylation, O-GlcNAc modification, which presents in HCC, EC, and pancreatic ductal adenocarcinoma (PDAC), also contributes to cancer stemness [8,41,42]. Hyper-O-GlcNAcylated eIF4E, induced by O-GlcNAc transferase (OGT)-endowed HCC cells with stem-like features, as exemplified by their higher proportion of CD133^+^ hepatoma cells, is a phenomenon possibly achieved through the physical interaction between eIF4E and the untranslated region of SOX2 5′ (5′UTR) [41]. In line with this, OGT knockout not only dramatically abrogated the self-renewal and tumorigenic capacities of ALDH^+^ esophageal CSCs, but also suppressed the tumor-initiating potential of the PC cell line S2VP10 in vivo [8,42]. Moreover, OGT prevented esophageal CSCs from cytotoxic CD8^+^T cell-mediated apoptosis for survival through a mechanism by which the exosomal OGT derived from esophageal CSCs was taken up by neighboring CD8^+^ T cells, thus leading to an upregulated PD-1 expression [8]. These data collectively implied the crucial role of OGT in sustaining the self-renewal and immune escape of esophageal CSCs [8]. As for PC, the O-GlcNAcylation of SOX2 catalyzed by OGT was required to maintain its transcriptional activity and stability for stemness phenotypes, whereas the OGT antagonist OSMI-1 efficiently delayed tumor progression by targeting the O-GlcNAcylated SOX2, alone with reduced OCT4 and NANOG levels in subcutaneous xenografts [42]. Intriguingly, Fuentes-García et al. revealed the opposite effect of O-GlcNAcylation on the stemness in CRC [43]. They found that the pharmacological targeting of OGT by Ac5SGlcNAc resulted in an enhanced spheroid formation ability through downregulating the global O-GlcNAcylation and upregulating the expression of the stemness markers CD133 and CD44 [43]. These findings suggest that O-GlcNAc modification may elicit distinct functions in a context-dependent manner.

### 4.3. Protein Methylation and Cancer Stemness

The hypermethylation of proteins induced by elevated methyltransferases has been well documented to contribute to aggressive cancer stemness across various GI cancers. In HCC, protein methyltransferase 6 (PRMT6) suppressed its cancer stemness properties through the methylation of CRAF on arginine 100, thus disrupting the binding between CRAF and RAS, which, in turn, suppressed the MEK/ERK axis [44]. Similar findings can be observed for esophageal CSCs, in which the highly expressed PRMT1 in esophageal squamous cell carcinoma (ESCC) catalyzed the histone H4R3 arginine asymmetric demethylation to activate the Wnt/β-catenin and Notch signaling pathways for stemness maintenance [45]. Furthermore, protein methylation is known to regulate CRC stemness. In ABHD5-deficient CRC cells, DPY30 translocated into the nucleus to potentiate SET1A-mediated YAP methylation at lysine 342 (Lys342), thus triggering a YAP-dependent c-Met overexpression to render stem-like features [46]. 

Apart from non-histone methylation, histone methylation is also essential for enhanced cancer stemness. Kryczek et al. discovered that the methylation of H3K79, governed by methyltransferase DOT1L, was required for the IL-22-dependent CRC stemness through the transactivation of STAT3 via IL-22 [47]. The higher level of either H3K79 or DOT1L was also closely associated with an unfavorable survival in patients with CRC [47]. In high-PCGF1-expressing CRC cells, PCGF1 increased histone H3K4me3 and reduced histone H3K27me3 at the promoter of CD133, CD44, and ALDH1A1 via the H3K4me3 methyltransferase KMT2A and the H3K27me3 demethylase KDM6A, respectively, which collaboratively upregulated the expression of these stemness markers [48]. Moreover, hepatic CSC-like LGR5^+^ cells were known to maintain their self-renewal and chemoresistance via the lysine-specific demethylase 1 (LSD1)-mediated mono- and di-methylation of histone H3 lysine 4 [11]. This methylation preferentially occurred at the promoters of β-catenin suppressors, such as Prickle1 and APC, which triggered β-catenin signaling activation and subsequent stem-like properties in HCC [11]. GASC1 was found to be overexpressed in ALDH^+^ ESCC cells in comparison to ALDH^−^ cells [49]. The depletion of GASC1 impaired ESCC stemness by methylating the NOTCH1 promoters H3K9me2 and H3K9me3 for NOTCH1 downregulation, a phenomenon that could be reversed by introducing NOTCH1 overexpression [49].

### 4.4. Protein Acetylation and Cancer Stemness

The imbalance between the acetylation and deacetylation of histone or non-histone proteins, as one of the important epigenetic modification processes, has been implicated in various forms of stemness-regulatory networking and is responsible for the maintenance of the self-renewal and other stem-like properties of CRC and HCC [50,51,52,53]. Using an iTRAQ-based proteomics analysis, Liu et al. identified that histone deacetylase 1 (HDAC1) represented the central roles of stem cell maintenance pathways, such as the Wnt and Notch pathways, and cell cycle regulation in the support of SOX4-dependent CRC stemness [50]. In addition to HDAC1-mediated deacetylation, the deacetylated histone H3 within the SIRT1/miR-1185-1/CD24 cascade also possessed a pivotal role in modulating CRC stemness [51]. Mechanistically, SIRT1 could deacetylate H3 lysine 9 on the promoter of miR-1185-1, which repressed the expression of miR-1185-1 and disrupted its targeting on the 3′UTR of CD24, thereby promoting CD24 translation and further enhancing stemness [51]. In HCC, the overexpression of HDAC11 was reported to impede histone acetylation at the promoter region of LKB1 to limit LKB1 transcription [52]. Subsequently, decreased LKB1 led to inhibited AMPK signaling and accelerated glycolysis, thus maintaining the stem-like population and promoting tumor progression in HCC [52]. Additionally, HDAC3-mediated deacetylation also contributed to HCC cancer stemness under glutamine deprivation [53]. Glutamine restriction stimulated Rictor/mTORC2 to induce HDAC3-mediated deacetylation and stabilize glutamine synthetase for an enhanced sphere formation, while targeting the mTORC2-HDAC3-glutamine synthetase cascade, which remarkably attenuated the self-renewal of CSCs and facilitated tumor elimination upon glutamine starvation therapy [53]. Taken together, these findings illustrated the central role of abnormal protein acetylation in sustaining cancer stemness, and targeting deacetylase might hold great therapeutic potential against GI cancers.

### 4.5. Others PTMs and Cancer Stemness

In addition to those common types mentioned above, other PTMs, such as ubiquitination, SUMOylation, lactylation, and palmitoylation, are also known to exert regulatory functions in GI cancer stemness [12,54,55,56,57,58,59]. For example, ectopically expressed FBXO11 facilitated Snail from ubiquitin-mediated proteasomal degradation, leading to a decreased expression of stemness markers, reduced sphere formation, and reduced ALDH1 activity in HCC cells [54]. Additionally, β-catenin ubiquitination, attenuated by Src homolog and collagen homolog 3 (Shc3), facilitated its nuclear translocation and the activation of the β-catenin/TCF pathway [12]. It subsequently stimulated the transactivation of MDR1 and stemness-related factors, including SOX2, OCT4, and NANOG, thereby enhancing HCC stemness and the resistance to sorafenib and doxorubicin [12]. Similarly, the sequential process of ubiquitination triggers stemness properties in CRC. The ubiquitination of TRIM21 E3 ubiquitin ligase through CSN6, in turn, reduced TRIM21-mediated OCT1 ubiquitination [55]. As a consequence, stabilized OCT1 contributed to ALDH1A1 upregulation and enhanced cancer stemness, implying the therapeutic potential of targeting serial ubiquitination in patients with CRC [55]. Highly expressed FBXL8 within CRC destabilized p53 via ubiquitination for enhanced tumorigenic and stemness properties, whereas a p53 loss restored the colony-forming ability and stemness markers suppressed by a FBXL8 knockdown [56].

Under hypoxic conditions, specific SUMO proteases 1 (SENP1) abrogated the deSUMOylation of HIF-1α at Lys391 and Lys477, thus improving its stability and transcription activity; deSUMOylated HIF-1α, together with HIF-2α and HIF-1β, in turn, transcriptionally upregulated SENP1 through binding to its promoter [57]. The positive feedback loop between SENP1 and HIF-1α expanded the HCC stemness, CD24^+^ subpopulation, and ultimate tumorigenesis, providing a promising target for HCC treatment [57]. Besides SUMOylation, hypoxia was also known to play a pivotal role in the maintenance of colon CSCs via the regulation of lactylation [58]. A study by Miao et al. found that glycolysis induced by hypoxia contributed to the lactylation of β-catenin, which subsequently stimulated the Wnt signaling pathway for enhanced CRC stemness properties, including the sphere-forming capacity and stemness markers (e.g., CD133 and Nanog), as well as cell viability [58]. Additionally, protein palmitoylation was involved in the palmitate (PA)-induced stemness phenotypes observed in liver CSCs [59]. PA facilitated the sphere-forming capability of liver CSCs, possibly through palmitoylating several proteins, such as α-enolase and c-Myc promoter-binding protein 1, etc., while palmitoylation inhibitors (tunicamycin and 2-bromohexadecanoic acid) could effectively repress this effect without affecting the cell viability [59].

## 5. The Crosstalk of Intracellular Protein PTMs on Stemness

Emerging evidence has suggested that there is a complex crosstalk between distinct protein PTMs for regulating the cancer stemness in GI cancers. The crosstalk between different types of PTMs has been identified to coordinate various stemness-related pathways for cancer stemness. For example, histone modifications, collaboratively caused by enhancers of zeste homolog 2 (EZH2)-mediated H3 lysine 27 trimethylation (H3K27me3) and the deacetylation of lysine residues on histone H3 and H4, contribute to REX1 downregulation and the subsequently enhanced self-renewal properties, as evidenced by the decreased frequency of TIC observed in REX1-overexpressing HCC xenografts [60]. Furthermore, a study by Wang et al. proved that the crosstalk between acetylation and ubiquitination allowed for the liver cancer stemness properties promoted by CRIP1 [61]. CRIP1 was responsible for the STUB1-BBOX1 interaction and STUB1-induced ubiquitination of BBOX1 at Lys240, which led to BBOX1 proteasomal degradation, reduced carnitine, and further inhibited β-catenin acetylation [61]. The deacetylated β-catenin then activated the transcription of stemness markers, including SOX2, OCT4, and NANOG, causing enhanced stem-like properties in HCC [61].

The complex crosstalk of PTMs in regulating GI cancer stemness highlights the need for a comprehensive understanding of the PTM landscape. Such knowledge may assist in developing novel, targeted therapies that can selectively target cancer stem cells and thus provide clinical benefits for patients with GI cancers. 

## 6. Therapeutic Targeting of PTMs in GI CSCs 

**Figure 2:**
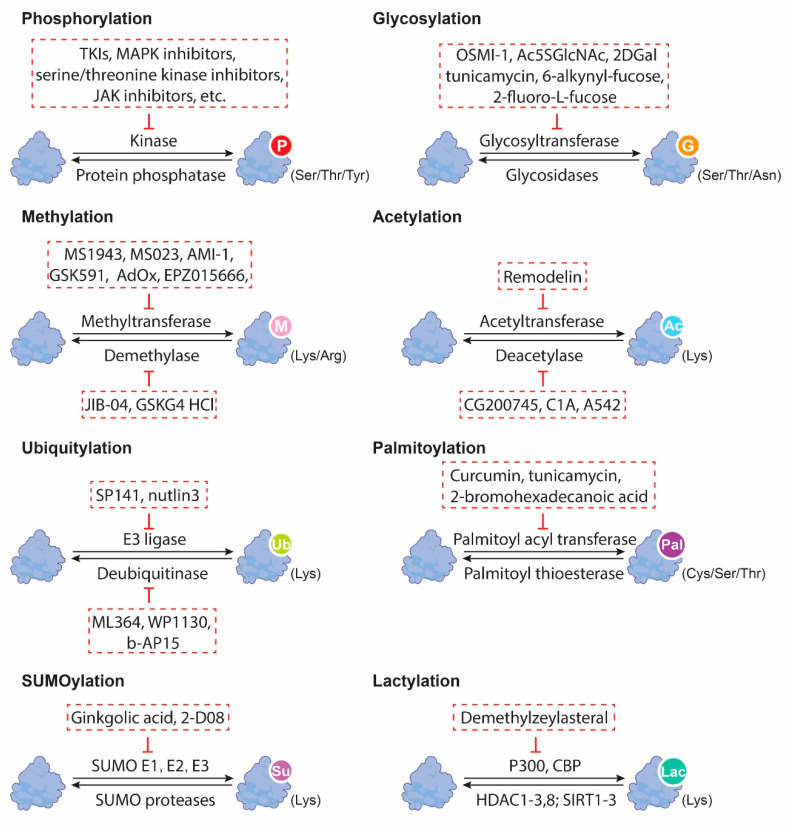
Small molecule inhibitors targeting distinct PTMs in GI cancers.

There is emerging knowledge that protein PTMs could serve as potential therapeutic targets for CSCs in GI cancers. CSCs are known to be resistant to conventional anticancer therapies and are thought to be responsible for tumor recurrence and metastasis. Targeting the PTMs in CSCs offers a promising opportunity to selectively eliminate these cells and improve GI patients’ outcomes (Figure 2). For example, targeting ubiquitination has been shown to reduce the stemness of cancer cells in CRC [56]. In addition, drugs that target the specific enzymes involved in PTM regulation, such as histone deacetylases, have been developed for the treatment of cancer and other diseases. Besides methyltransferases, aspirin was also capable of methylating histone 3 (H3) for stemness inhibition in a COX-1-independent process [62]. Using a high-throughput siRNA platform, aspirin was demonstrated not only to induce H3 hypermethylation, as manifested by the elevated levels of H3 trimethylation markers, including H3K4-3Me, H3K9-3Me, H3K27-3Me, H3K36-3Me, and H3K79-3Me, but also to suppress the expression of inflammation-related stemness gene treatments, especially ICAM3, through decreasing the histone demethylase (KDM6A/B) [62]. As such, aspirin administration could inhibit the ALDH^+^ proportion and sphere-forming capacity, increase the sensitivity in vitro, and suppress tumor development [62]. Moreover, the co-administration of aspirin and KDM6A/B or ICAM3 inhibitors effectively delayed tumor progression in vivo, suggesting a combinatorial strategy for cancer treatment [62]. JIB-04, as an inhibitor of histone demethylases, functions by enhancing the methylation of H3K9 and H3K36 to disrupt the Wnt/β-catenin signaling for the impaired tumor-initiating properties of colorectal CSCs [63]. Demethylzeylasteral (DML) has been demonstrated to block histone lactylation via two modification sites, H3K9la and H3K56la, by decreasing the lactic acid in liver CSCs [64]. Functionally, DML treatment led to a reduction in stemness markers, such as CD133 and CD44, cell viability in vitro, and xenografts growth in vivo [64].

**Table 1:** The regulatory mechanisms of non-histone protein posttranslational modifications in GI cancer stemness.

Cancer Type	PTM Types	Proteins Targets	Mechanisms	Ref
EC	Phosphorylation	STAT3	Phosphorylation of STAT3 induces NANOG upregulation.	[16]
		p53	Nuclear phosphorylated p53 bound to NANOG promoter to inhibit OCT4- and SOX2-induced NANOG transcription.	[34]
	Glycosylation	--	OGT depletion attenuated the self-renewal and tumorigenic capacities of ALDH^+^ esophageal CSCs.	[42]
HCC	Deacetylation	β-catenin	Deacetylation of β-catenin caused by BBOX1 degradation transactivated SOX2, OCT4, and NANOG.	[61]
	Glycosylation	--	Knockdown of glycosylation-associated genes (e.g., CAD, SLC51B, LGALS3, B3GAT3, and MT3) led to decreased stemness markers CD24, CD44, CD20, FOXM1, and EpCAM.	[37]
		CD147, ICAM-1, EGFR, EPHA2	Under glucose restriction, FUT1-induced α1,2-fucosylation on CD147, ICAM-1, EGFR, and EPHA2 dysregulated AKT/mTOR/4EBP1 signaling to potentiate stemness.	[10]
		eIF4E	O-GlcNAcylatd eIF4E directly interacted with 5′UTR of SOX2 to result in an enrichment of CD133^+^ hepatoma cells.	[41]
	Methylation	CRAF	PRMT6 methylated CRAF on arginine 100 to suppress the RAS/RAF binding and MEK/ERK axis.	[44]
	Palmitoylation	α-enolase, c-Myc promoter-binding protein 1, etc.	Palmitate stimulated the palmitoylation of these proteins for enhanced sphere-forming capability.	[59]
	SUMOylation	HIF-1α	SUMO protease SENP1 and HIF-1α formed a positive feedback loop to expand CD24^+^ subpopulation.	[57]
PC	Glycosylation	--	ST6Gal1 induced stem cell transcription factors Sox9 and Slug.	[40]
		--	Knockout of OGT inhibited the tumor-initiating capacity.	[8]
		SOX2	O-GlcNAcylation of SOX2 at S246A by OGT increased its stability in the nucleus.	[42]
CRC	Phosphorylation	YAP	Dephosphorylated YAP contributed to stiff-matrix-potentiated CRC stemness features.	[18]
	Methylation	YAP	Methylation of YAP at K342 induced c-Met upregulation for stemness features.	[46]
	Ubiquitination	p53	p53 ubiquitination caused p53 destabilization and further stemness features.	[56].
	Glycosylation	--	FUT9-mediated hyperfucosylation also triggered Sox2, ALDH, and CD44 expression and tumor sphere formation.	[9]
		Fas receptors	ST6Gal1 loss reduced the CD133^+^/ALDH1^+^ CSCs population.	[7]
		--	Global O-GlcNAcylation downregulation led to increased CD133 and CD44 expression and enhanced spheroid-forming ability.	[43]
	Lactylation	β-catenin	β-catenin lactylation stimulated the Wnt signaling pathway to potentiate CRC stemness.	[58]
GC	Phosphorylation	YAP	SCD1 induced YAP phosphorylation to affect cell stemness.	[29]

**Table 2:** The regulatory mechanisms of histone protein posttranslational modifications in GI cancer stemness.

Cancer Type	PTM Types	Proteins Targets	Mechanisms	Ref
EC	Methylation	NOTCH1	Demethylated NOTCH1 at its promoters H3K9me2 and H3K9me3.	[49]
		histone H4	Demethylation of histone H4 arginine catalyzed by PRMT1 activated Wnt/β-catenin and Notch signaling pathways.	[45]
		H3K9me2, H3K9me3	GASC1 methylated H3K9me2 and H3K9me3 on NOTCH1 promoter to reduce NOTCH1, leading to impaired stemness.	[49]
HCC	Methylation	Prickle1, APC	Methylation of Prickle1 and APC promoters activated β-catenin signaling and subsequent stem-like characteristics.	[11]
	Deacetylation	LKB1	HDAC11 mediated histone deacetylation at the promoter region of LKB1 to inhibit LKB1 transcription, which further abrogated AMPK signaling and accelerated glycolysis to maintain CSCs.	[52]
CRC	Methylation	H3K79	H3K79 methylation contributed to IL-22-induced transcriptional upregulation of STAT3 and subsequent stemness features.	[47]
		H3K4me3, H3K27me3	Upregulated histone H3K4me3 and downregulated histone H3K27me3 collaboratively induced transactivation of CD133, CD44, and ALDH1A1 by binding to their promoters.	[48]
	Acetylation	H3 lysine 9	SIRT1 deacetylated H3 lysine 9 on miR-1185-1 promoter to repress its expression and disrupted its targeting on CD24 3′UTR.	[51]

Abnormal protein PTMs have been demonstrated to affect the efficacy of oncotherapy via the modulation of cancer stemness. In irinotecan-resistant colon carcinoma, there was a higher percentage of CD133^+^/ALDH1^+^ CSCs, accompanied by an elevation in the ST6Gal-I level and Fas receptors α2,6-sialylation, whereas the depletion of ST6Gal-I dramatically reduced the enriched CSC subpopulation within the chemoresistant cells [7]. Furthermore, EC xenografts were found to exhibit a higher OGT level upon 5-FU treatment [8]. OGT could further contribute to the immune tolerance of esophageal CSCs, as exosomal OGT secreted from esophageal ALDH^+^ CSCs could enter into the surrounding CD8^+^ T cells to upregulate the PD-1 expression [8]. Moreover, highly expressed FUT9 was responsible for preventing murine colon adenocarcinoma MC38 cells bearing stem-like phenotypes from 5-FU-induced cell death [9]. A blockade of α1,2-fucosylation using 2DGal was found to eradicate TICs and sensitized HCC tumors towards sorafenib [10]. The mono- and di-methylation of histone H3 lysine 4, mediated by LSD1, was responsible for the maintenance of the self-renewal properties and resistance to cisplatin and sorafenib in hepatic Lgr5^+^ cells [11]. In addition, the deubiquitylation of β-catenin accelerated its nuclear translocation and the activation of the β-catenin/TCF pathway, which further transcriptionally upregulated MDR1 and its stemness markers to confer resistance to sorafenib and doxorubicin [12]. TRIM25 stabilized H3K27me3 methyltransferase EZH2 by blocking TRAF6-mediated ubiquitination for EZH2 degradation, thus maintaining the stem-like properties for an enhanced oxaliplatin resistance in CRC [13]. The genetic knockdown or pharmacological inhibition of EZH2 sensitized CRC towards oxaliplatin, revealing a promising strategy for overcoming therapy resistance [13].

The development of new, targeted therapies that can selectively target cancer stem cells by modulating PTMs is an exciting area of research with significant therapeutic potential for patients with gastrointestinal cancers. To date, more research is needed to elucidate the specific PTMs that are critical for regulating GI cancer stemness and to develop effective drugs based on these altered PTMs.

## 7. Conclusions and Future Perspectives

Mounting evidence has demonstrated the crucial role of protein PTMs in regulating cancer stemness in GI malignancies. The different types of PTMs, including phosphorylation, glycosylation, methylation, acetylation, and ubiquitination, have been shown to regulate the key signaling pathways involved in cancer stemness, such as the Wnt/β-catenin, Notch, and Akt pathways. Moreover, PTMs are required to affect the activity of the transcription factors involved in the cancer stemness and metabolism of CSCs. Targeting PTMs holds promise for selectively eliminating CSCs, which are often resistant to conventional therapies, and could thereby improve patients’ clinical outcomes. However, developing PTM-based cancer treatment strategies for targeting CSCs presents significant challenges. Firstly, apart from these well-characterized PTM types, our current knowledge regarding the functional roles of several rare PTMs, such as S-glutathionylation and nitrosylation, remains understudied in GI cancers. For example, targeting GSTP1, an enzyme that catalyzes S-glutathionylation, has been demonstrated to diminish stemness properties and sensitize lung cancer towards tyrosine kinase inhibitors, suggesting the potential role of GSTP1-mediated glutathionylation in potentiating cancer stemness [5]. Secondly, the crosstalk between different types of PTMs and their effects on GI cancer stemness is a complex and dynamic process that requires further investigation. Additionally, the protein modifications in CSC subpopulations within different tissue contexts have been found to be heterogeneous and plastic. Moreover, there is a lack of evidence evaluating the efficacy and safety of targeting these PTMs in patient-derived models and preclinical studies for GI cancer treatment. Further studies should focus on elucidating the roles of specific PTMs in cancer stemness, identifying specific targets, and designing drugs that can effectively target these molecules. The development of technologies that can accurately detect and analyze PTMs in vivo will also be important for the translation of PTM-based therapies into the clinic.

In summary, developing effective PTM-based therapies will require a comprehensive knowledge of the complex regulatory networks involved in cancer stemness, which could assist in selectively eliminating CSCs while minimizing damage to normal cells. A better understanding of the function of diverse PTMs in GI cancer stemness can not only provide therapeutic strategies for cancer treatments, but also overcome therapy resistance, thus possessing clinical benefits in the future.

## Data Availability

Not applicable.
